# Supplementary material for: A whole blood assay for antibody dependent phagocytosis of Plasmodium falciparum infected erythrocytes
Source: Commun Med (Lond). 2025 Jul 7;5:277. doi: 10.1038/s43856-025-00989-2 (PMC12234751; doi:10.1038/s43856-025-00989-2)
Supplement: Supplementary file 2 — Supplementary Information [file 43856_2025_989_MOESM2_ESM.pdf]

SUPPLEMENTARY TABLES AND FIGURES

Supplementary Table 1 Demographic and clinical data of Malawian pregnant women at enrolment (16-28 weeks of gestation)

	Primigravida<br>n=20	Multigravida<br>n=20
Mean age [SD] years	18.2 [2.5]	23.2 [3.7]
Residence		
Rural	19 (95.0)	20 (100.0)
Non-rural	1 (5.0)	0 (0.0)
Bed net use	18 (90.0)	17 (85.0)
P. falciparum by light microscopy	6 (30.0)	2 (10.0)
P. falciparum by PCR	14 (70.0)	6 (30.0)
Haemoglobin levels g/dL, mean (SD)	10.08 [1.07]	11.96 [0.98]

Data are n (%) unless otherwise stated. Abbreviation: SD, standard deviation.

Supplementary Table 2 Demographic and clinical data of pregnant Papua New Guinean women at enrolment (14-26 weeks of gestation) and delivery

	Non-placental malaria (non-PM) n=27	Placental malaria (PM) n=50
ENROLMENT		
Mean age [SD] years	23.1 [4.4]	24.0 [5.0]
Maternal gravidity		
Primigravidae	14 (51.9)	29 (58.0)
Secundigravidae	8 (29.6)	7 (14.0)
Multigravidae	5 (18.5)	14 (28.0)
Residence		
Rural	18 (66.7)	38 (76.0)
Non-rural	9 (33.3)	12 (24.0)
Bed net use	21 (77.8)	40 (80.0)
P. falciparum by light microscopy	4 (14.8)	4 (8.0)
P. falciparum by PCR	5 (18.5)	5 (10.0)
IPTp regime		
SPAZ	9 (33.3)	20 (40.0)
SPCQ	18 (66.7)	30 (60.0)
DELIVERY		
P. falciparum by light microscopy in placental blood	0 (0)	50 (100)
P. falciparum by PCR in peripheral blood	13 (48.1)	21.0 (42)
P. falciparum by PCR in placental blood*	10 (43.4)	10.0 (23.8)
Histology#		
P. falciparum acute infection	0	20 (40.0)
P. falciparum chronic infection	0	30 (60.0)
P. falciparum past infection	12 (44.4)	0
P. falciparum uninfected	15 (55.6)	0

*Missing placental PCR data of pregnant women (PM=8 and non-PM=4) (Adapted from (Aitken et al., 2021)).

Acute infection: parasites without malaria pigment in leukocytes or fibrin deposits; chronic infection: parasites and malaria pigment in leukocytes or fibrin deposits; past infection: no parasites, pigment in leukocytes and/or fibrin deposits; uninfected: neither parasites nor malaria pigment detected.

Data are n (%) unless otherwise stated. Abbreviations: SD, standard deviation; PCR, polymerase chain reaction; SPAZ, Sulphadoxine-pyrimethamine and azithromycin; SPCQ, Sulphadoxine-pyrimethamine + chloroquine.

Supplementary Table 3. Pairwise correlations between monocyte and neutrophil phagocytosis using isolated cells and in whole blood

	Purified monocytes*	Purified neutrophils*	Whole blood monocytes	Whole blood neutrophils
Purified monocytes*	1.00			
Purified neutrophils*	0.40 (0.0003)	1.00		
Whole blood monocytes	0.20 (0.08)	0.10 (0.69)	1.00	
Whole blood neutrophils	0.16 (0.17)	0.10 (0.41)	0.90 (<0.0001)	1.00

Samples from 77 Papua New Guinean women were run in all assays. Spearman correlations (and P values). *Data from Aitken et al (2021) eLife *10:e65776.*

Supplementary Table 4 Demographic and clinical data of children with severe malaria at presentation and convalescence (8 weeks later)

Characteristic	Severe malaria		Uncomplicated malaria	
	Acute (n=211)	Convalescent (n=86)	Acute (n=79)	Convalescent (n=86)
Age in months, median (IQR)	43 (30-60)	42 (30-58)	47 (31-59)	47 (31-60)
Sex (%)				
Female	44.8	44.3	42.6	42.4
Male	55.2	55.7	57.4	57.6
Haemoglobin at presentation, median (IQR) g/L	79 (55-91)	N/A	89 (72-100)	N/A
P. falciparum parasitaemia/µl at presentation, median (IQR)	86,171 (27,064-181,072)	N/A	21,938 (4,811-66,053)	N/A

Abbreviations: IQR, Interquartile range; N/A, Not Applicable

SUPPLEMENTARY FIGURES

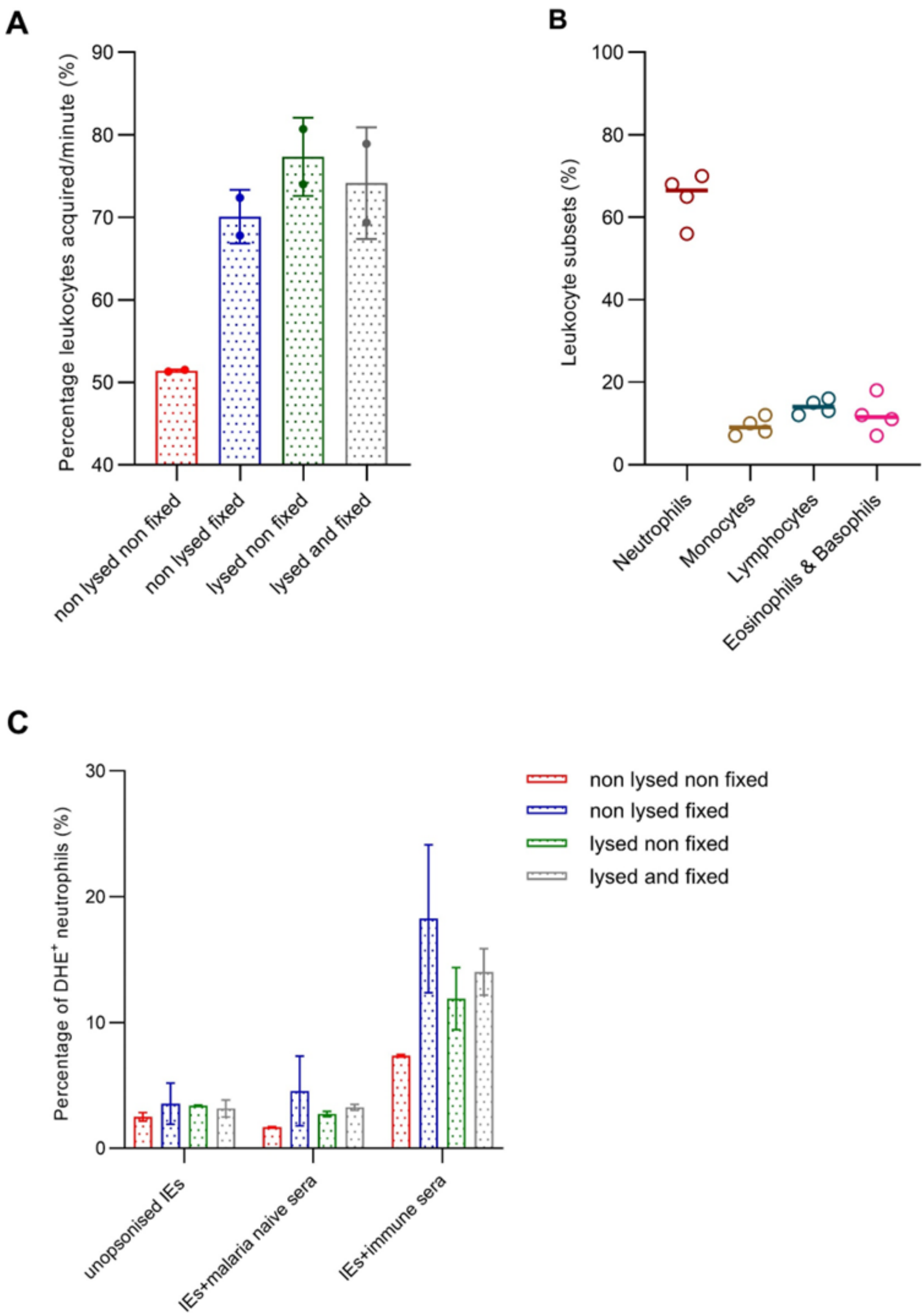

Supplementary Figure 1. The effects of erythrocyte lysis and fixation on the total percentage of leukocytes, the differential leukocyte populations (%) following lysis and fixation, and the percentage of DHE+ neutrophils per each testing condition. (A). The rate of acquisition of leukocytes (%) when whole blood was neither lysed nor fixed (red); not lysed but fixed (blue); lysed but not fixed (green); and both lysed and fixed (grey). (B)

Identification of leukocyte subsets by fluorescently labelled antibodies against CD14, CD16, and CD66b in lysed and fixed preparations from four different whole blood donors from Melbourne. In (C), the percentage of DHE-positive neutrophils when whole blood was neither lysed nor fixed (red) ; not lysed but fixed (blue); lysed but not fixed (green); and both lysed and fixed (grey). IEs were unopsonised; opsonised with malaria naïve sera or opsonised with immune sera. The results are plotted as the mean and standard error of the mean from two independent experiments conducted in triplicates in (A) and (C).

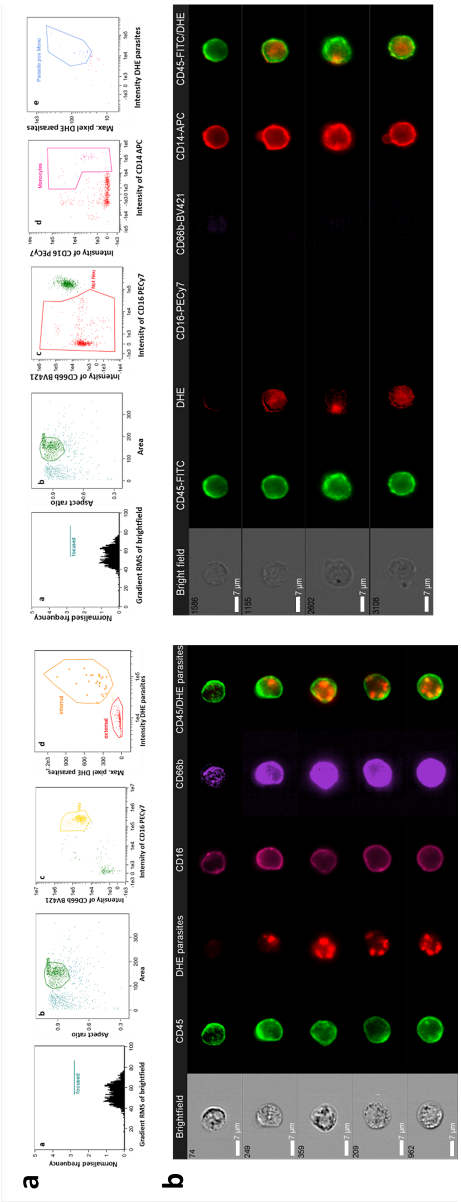

Supplementary Figure 2 Imagestream analysis of IE colocalization with neutrophils and monocytes in whole human blood. (A) gating strategy for identifying parasites internalised by neutrophils and monocytes by imaging flow cytometry. The CD45+ leukocytes in the best focus were gated based on gradient RMS. An RMS value above 50 is usually considered to be in-focus. An aspect ratio value closer to one symbolised that the cells are circular, while the ratios between 0.4 to 0.8 show that they would be cell aggregates and doublets. All single-cell neutrophils are gated based on their expression of both CD66b and CD16 fluorescent positivity while all single-cell monocytes were identified based on their CD14 and CD16 positivity. Internalised parasites were selected using an internalisation score (a score of > 0.3 were considered to have internalised particles, and those with a score of < 0.3 were supposed to have surface-bound particles). Antihuman CD45 is used as a membrane marker (green) to distinguish between internalised versus external parasites (red). A total of 2500 single leukocytes in focus were collected for the analyses.

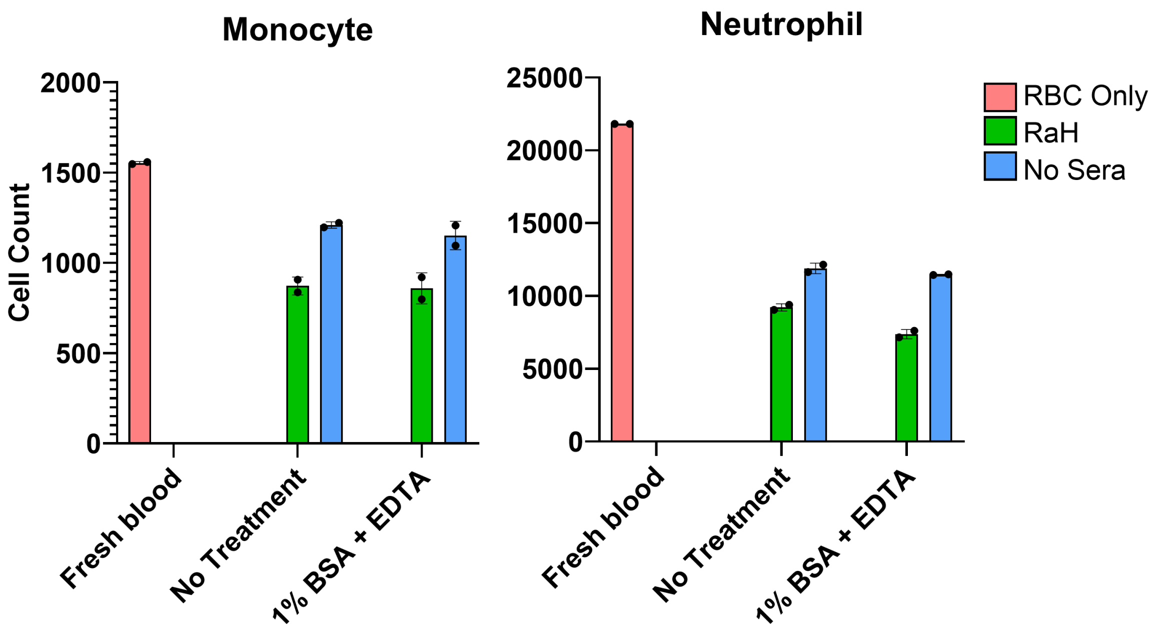

Supplementary Figure 3. Effect of incubation on cell counts. To determine whether monocytes or neutrophils were differentially depleted using the phagocytosis experiments we diluted fresh blood 1:1 with RPMI or incubated it with IEs that were not opsonised (“No sera”) or opsonised with rabbit antihuman erythrocyte antibody (RaH). For IEs, wells were untreated (“No treatment”) or blocked with 1% BSA + EDTA. Decrease in subsequent cell numbers was broadly similar for monocytes (left) and neutrophils (right), and not affected by pre-blocking of wells.

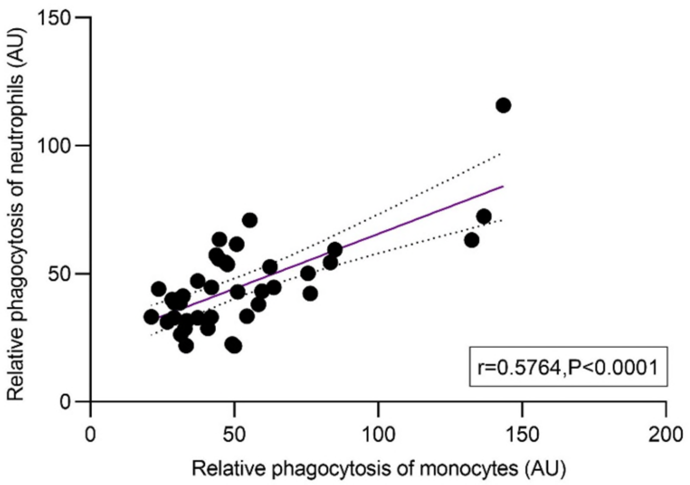

Supplementary Figure 4 Correlation between neutrophil and monocyte phagocytosis of *P. falciparum*-IEs when opsonised with malaria-exposed plasma of pregnant women from Malawi in Figure 2C. The purple line running diagonally indicates the direction of the linear relationship between the x and y-axes. The dotted lines on either side of the purple line represent 95% confidence intervals. The linear relationship was calculated using the Spearman correlation coefficient (AU=Arbitrary units).

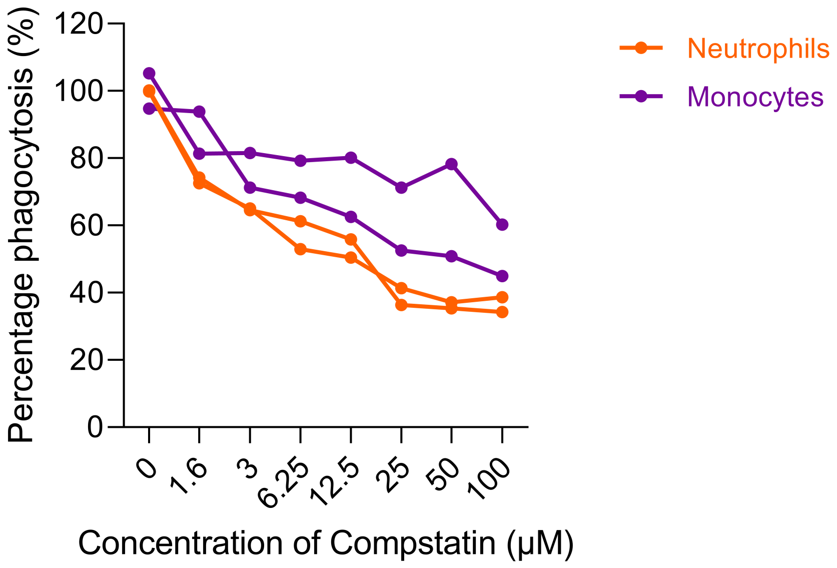

Supplementary Figure 5. The inhibition of complement activation decreases neutrophil and monocyte phagocytosis of antibody-opsonised IEs in peripheral whole blood. The titration curve for the dose-response of compstatin on ADP of *P. falciparum*-IEs by neutrophils and monocytes in whole blood. The differences in phagocytosis of immune plasma opsonised IEs by neutrophils and monocytes in compstatin-added whole blood relative to whole blood with no compstatin added (compstatin=0 µM) are plotted. The results are expressed as the mean and the standard error of the mean from two independent experiments conducted in duplicates.

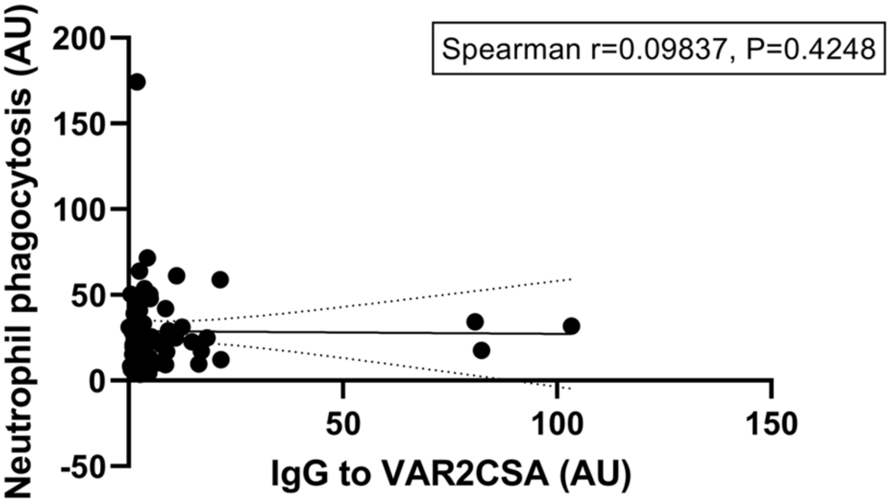

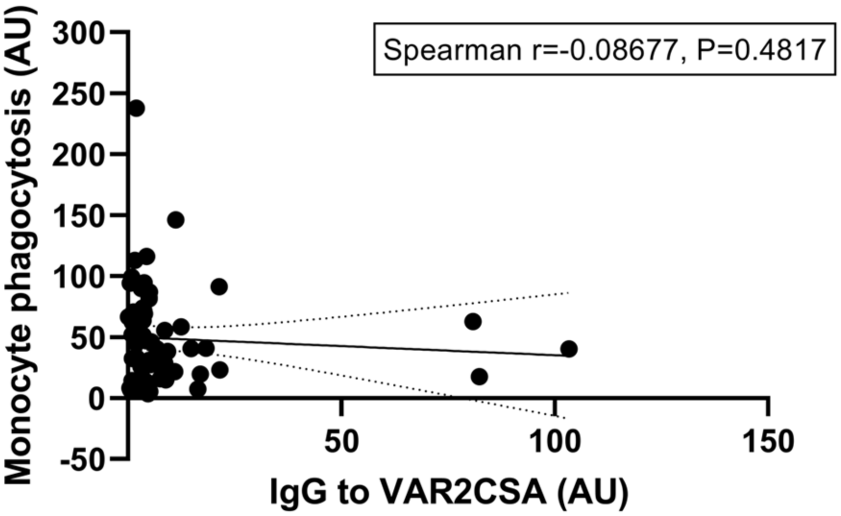

Supplementary Figure 6 Correlation between neutrophil (top) and monocyte (bottom) phagocytosis of *P. falciparum*-IEs with IgG to VAR2CSA. The solid black line indicates the linear relationship between the x and y-axes. The dotted lines on either side represent 95% confidence intervals. Antibody to full length FCR3 VAR2CSA was measured by ELISA. The linear relationship was calculated using the Spearman correlation coefficient (AU=Arbitrary units).
