# Supplementary material for: A whole blood assay for antibody dependent phagocytosis of Plasmodium falciparum infected erythrocytes
Source: Commun Med (Lond). 2025 Jul 7;5:277. doi: 10.1038/s43856-025-00989-2 (PMC12234751; doi:10.1038/s43856-025-00989-2)
Supplement: Supplementary file 3 — Description of Additional Supplementary Files [file 43856_2025_989_MOESM3_ESM.docx]

**Description of Additional Supplementary Files**

File name- Supplementary Data 1

File description - Dataset underlying figures in the manuscript
