# Supplementary material for: A whole blood assay for antibody dependent phagocytosis of Plasmodium falciparum infected erythrocytes
Source: Commun Med (Lond). 2025 Jul 7;5:277. doi: 10.1038/s43856-025-00989-2 (PMC12234751; doi:10.1038/s43856-025-00989-2)
Supplement: Supplementary file 5 — Reporting summary [file 43856_2025_989_MOESM5_ESM.pdf]

nature portfolio

Corresponding author(s): Stephen Rogerson

Last updated by author(s): 2024/08/30

Reporting Summary

Nature Portfolio wishes to improve the reproducibility of the work that we publish. This form provides structure for consistency and transparency in reporting. For further information on Nature Portfolio policies, see our Editorial Policies and the Editorial Policy Checklist.

Statistics

For all statistical analyses, confirm that the following items are present in the figure legend, table legend, main text, or Methods section.

n/a	Confirmed	
☐	☒	The exact sample size (*n*) for each experimental group/condition, given as a discrete number and unit of measurement
☐	☒	A statement on whether measurements were taken from distinct samples or whether the same sample was measured repeatedly
☐	☒	The statistical test(s) used AND whether they are one- or two-sided<br>*Only common tests should be described solely by name; describe more complex techniques in the Methods section.*
☒	☐	A description of all covariates tested
☒	☐	A description of any assumptions or corrections, such as tests of normality and adjustment for multiple comparisons
☐	☒	A full description of the statistical parameters including central tendency (e.g. means) or other basic estimates (e.g. regression coefficient) AND variation (e.g. standard deviation) or associated estimates of uncertainty (e.g. confidence intervals)
☒	☐	For null hypothesis testing, the test statistic (e.g. *F*, *t*, *r*) with confidence intervals, effect sizes, degrees of freedom and *P* value noted<br>*Give P values as exact values whenever suitable.*
☒	☐	For Bayesian analysis, information on the choice of priors and Markov chain Monte Carlo settings
☒	☐	For hierarchical and complex designs, identification of the appropriate level for tests and full reporting of outcomes
☒	☐	Estimates of effect sizes (e.g. Cohen's *d*, Pearson's *r*), indicating how they were calculated

Our web collection on statistics for biologists contains articles on many of the points above.

Software and code

Policy information about availability of computer code

Data collection	Data collected in FlowJo, exported to Microsoft Excel
Data analysis	Data analysed with Graphpad Prism

For manuscripts utilizing custom algorithms or software that are central to the research but not yet described in published literature, software must be made available to editors and reviewers. We strongly encourage code deposition in a community repository (e.g. GitHub). See the Nature Portfolio guidelines for submitting code & software for further information.

Data

Policy information about availability of data

All manuscripts must include a data availability statement. This statement should provide the following information, where applicable:

- Accession codes, unique identifiers, or web links for publicly available datasets
- A description of any restrictions on data availability
- For clinical datasets or third party data, please ensure that the statement adheres to our policy

Data underlying figures provided as supplementary file

Human research participants

Policy information about studies involving human research participants and Sex and Gender in Research.

Reporting on sex and gender	Experiments were performed with parasites that cause placental malaria, or cause severe malaria in young children. The children with severe malaria were recruited without consideration of gender.
Population characteristics	Relevant data are presented in Tables S1, S2 and S3
Recruitment	Pregnant women were recruited in second trimester to a malaria prevention study if they had no medical contraindication to participation and provided written informed consent. Children were recruited at hospital presentation with severe or uncomplicated malaria if parents or guardians provided written informed consent. All children with severe malaria were eligible, children with uncomplicated malaria represented an unselected convenience sample.
Ethics oversight	The study of pregnant women was approved by the PNG Institute of Medical Research Institutional Research Board, the PNG Medical Research Advisory Council, and the Melbourne Health Human Research Ethics Committee. The study in children was approved by the PNG Institute of Medical Research's Institutional Review Board (IRB No.1103) and the Medical Research Advisory Council of the PNG Department of Health

Note that full information on the approval of the study protocol must also be provided in the manuscript.

Field-specific reporting

Please select the one below that is the best fit for your research. If you are not sure, read the appropriate sections before making your selection.

☒ Life sciences ☐ Behavioural & social sciences ☐ Ecological, evolutionary & environmental sciences

For a reference copy of the document with all sections, see nature.com/documents/nr-reporting-summary-flat.pdf

Life sciences study design

All studies must disclose on these points even when the disclosure is negative.

Sample size	For pregnant women, all participants with peripheral blood parasitaemia without placental malaria (N=27) were included, and 50 matched women with placental malaria. Samples were selected for a previous Systesm Serology study (Aitken et al, eLife 2021). For children all available remaining samples were used from a case-control study (Manning et al, Plos One 2011, 6(12):e29203).
Data exclusions	No data were excluded.
Replication	Experiments were replicated as described in individual figure legends.
Randomization	Randomization is not relevant to the present study. Clinical presentation was used to define distinct groups.
Blinding	Experiments were performed blind to specific condition or (for patient samples) clinical grouping.

Reporting for specific materials, systems and methods

We require information from authors about some types of materials, experimental systems and methods used in many studies. Here, indicate whether each material, system or method listed is relevant to your study. If you are not sure if a list item applies to your research, read the appropriate section before selecting a response.

Materials & experimental systems

n/a	Involved in the study	
☐	☒	Antibodies
☒	☐	Eukaryotic cell lines
☒	☐	Palaeontology and archaeology
☒	☐	Animals and other organisms
☐	☒	Clinical data
☒	☐	Dual use research of concern

Methods

n/a	Involved in the study	
☒	☐	ChIP-seq
☐	☒	Flow cytometry
☒	☐	MRI-based neuroimaging

Antibodies

Antibodies used	FITC antihuman CD14 (BioLegend Cat no. 301804), PECy7 antihuman CD16 (BD Biosciences cat no. 557744), BV421 antihuman CD66b (Beckton Dickinson Cat no. 562940), and FITC antihuman CD45 (BD Biosciences cat no.11-0459-42).
Validation	These are all widely-used commercial antibodies to common antigens on specific leukocyte types for flow cytometric determination of cell populations

Clinical data

Policy information about clinical studies

All manuscripts should comply with the ICMJE guidelines for publication of clinical research and a completed CONSORT checklist must be included with all submissions.

Clinical trial registration	The samples from pregnant women were from a subset of participants in a clinical trial of malaria prevention ClinicalTrials.gov NCT01136850. Samples from children were collected as part of case-control studies of severe malaria and no intervention was tested.
Study protocol	*Note where the full trial protocol can be accessed OR if not available, explain why.*
Data collection	Pregnant women: November 2009- August 2012. Children: October 2006-December 2009. Both at health facilities in Madang Province, Papua New Guinea.
Outcomes	Not relevant to this use of samples

Flow Cytometry

Plots

Confirm that:

☒ The axis labels state the marker and fluorochrome used (e.g. CD4-FITC).

☒ The axis scales are clearly visible. Include numbers along axes only for bottom left plot of group (a 'group' is an analysis of identical markers).

☒ All plots are contour plots with outliers or pseudocolor plots.

☒ A numerical value for number of cells or percentage (with statistics) is provided.

Methodology

Sample preparation	Trophozoite stage P faciparum infected red blood cells were separated using Percoll-gradient purification.The purity of trophozoite stage parasites after Percoll-gradient purification was assessed using Giemsa stain. Parasite smears on glass slides were Giemsa stained, Methanol fixed, air dried, and observed under a light microscope. The purity was determined as a percentage of Giemsa-positive trophozoites to total RBCs. The purity of trophozoites was between 90-98%. They were opsonised with plasma samples from pregnant women from PNG or children from Malawi. The antibody opsonised P. falciparum-IEs were mixed with healthy human blood to reach a final dilution of 1:4 for human blood in a 96-U bottom plate. The plates were incubated at 37oC with 5% CO2 in an incubator for cell-mediated phagocytosis. The phagocytosis was stopped via centrifugation of the plates at 350 x g for five minutes at 4oC. The pellets were labelled with fluorescent antibodies against neutrophil and monocyte cell membrane markers. Following incubation on ice in the dark for 30 minutes, the plates were spun down at 350 x g for 5 minutes at 4oC to collect the pellet. The RBCs were lysed using 1X FACS lysing solution. Upon lysis, the cells were fixed with 2% PFA in PBS and resuspended in cold FACS buffer until acquired by a flow cytometer.
Instrument	CytoFLEX S or CytoFLEX LX, both Beckman Coulter
Software	CytExpert for Data Acquisition in CytoFLEX S or CytoFLEX LX and FlowJo for Data Analysis
Cell population abundance	The percentages of different leukocyte subsets following whole blood lysis and leukocyte fixation in healthy human volunteers are 65% (±6%) of neutrophils, 9% (±2%) of monocytes, 15% (±1%) of lymphocytes, and 11% (±4%) of eosinophils and basophils. These cell populations were identified using their differences in forward and side light scatter properties and fluorescently labelled antibodies against CD14, CD16, and CD66b by flow cytometry.
Gating strategy	The leukocyte population was first identified using differences in light scatter properties of leukocytes and red blood cells using violet (SSC-A-violet) and blue (SSC-A-blue) side scatter. The neutrophil and monocyte populations were separately identified by the differences in their forward (FSC-A) and side (SSC-A) scatter. The doublet neutrophils and monocytes were removed using a single cell gating (FSC-H vs. FSC-A). The single neutrophils were confirmed using cell surface markers, anti-CD66b antibody conjugated to BV421, and anti-CD16 conjugated to PECy7. Single monocytes were confirmed using CD14 conjugated to FITC and CD16 conjugated to PECy7 (D). The dihydroethidium (DHE)-labelled Plasmodium falciparum-IEs that were associated with the neutrophils or monocytes were gated based on the negative population of unopsonised IEs.

Gating is shown in Figure 1 of the manuscript.

☒ Tick this box to confirm that a figure exemplifying the gating strategy is provided in the Supplementary Information.
